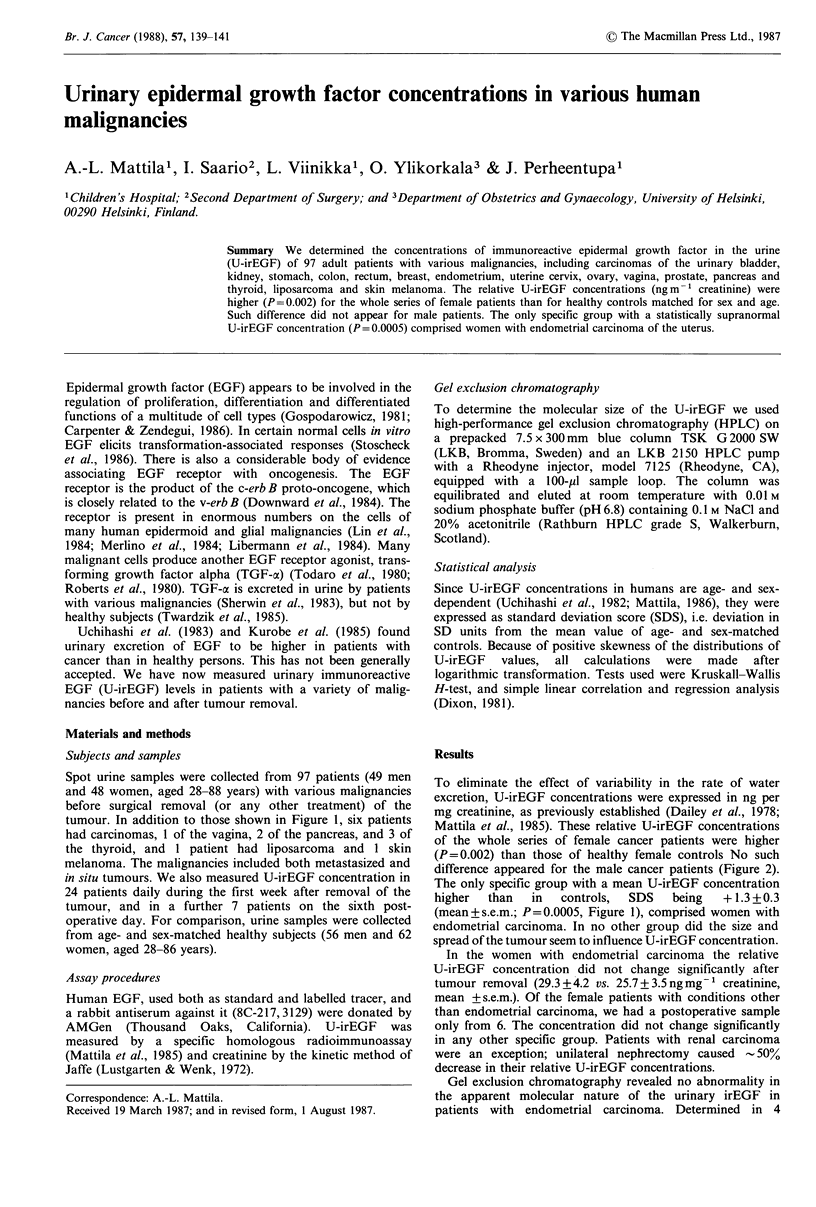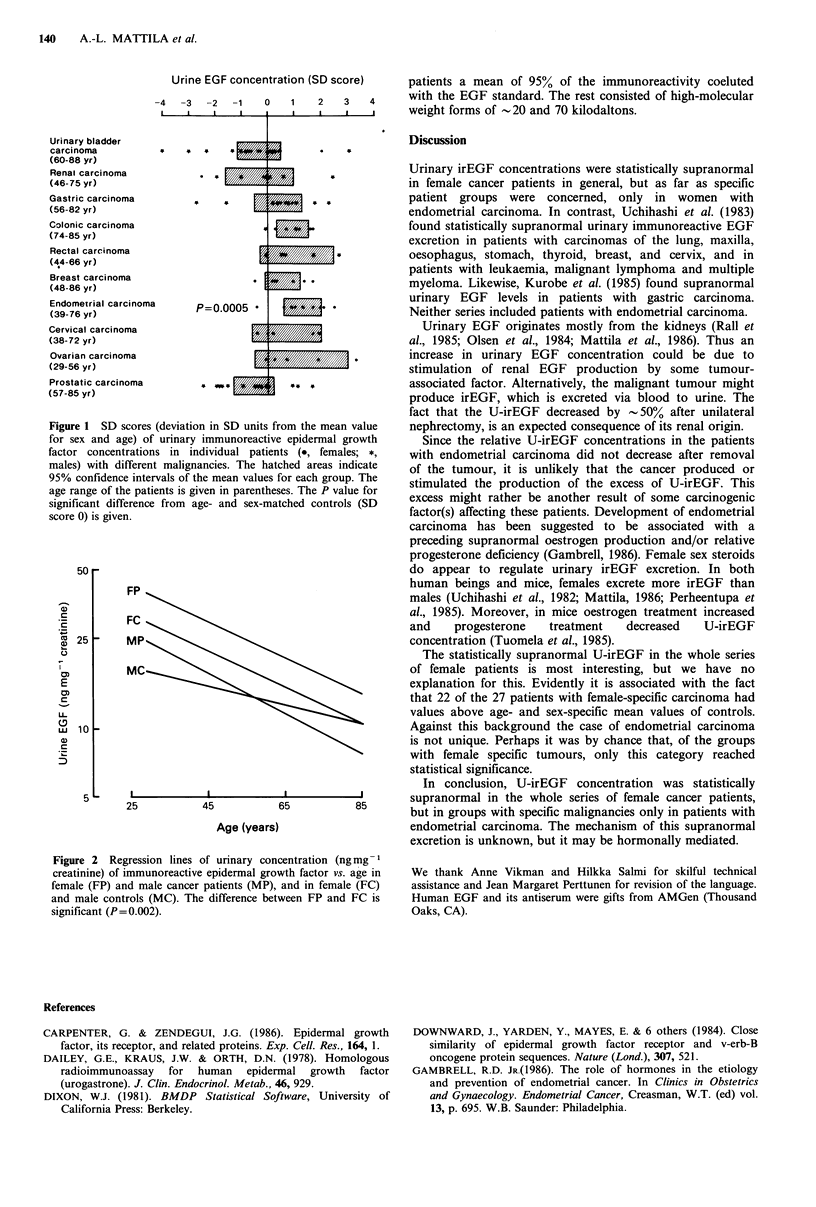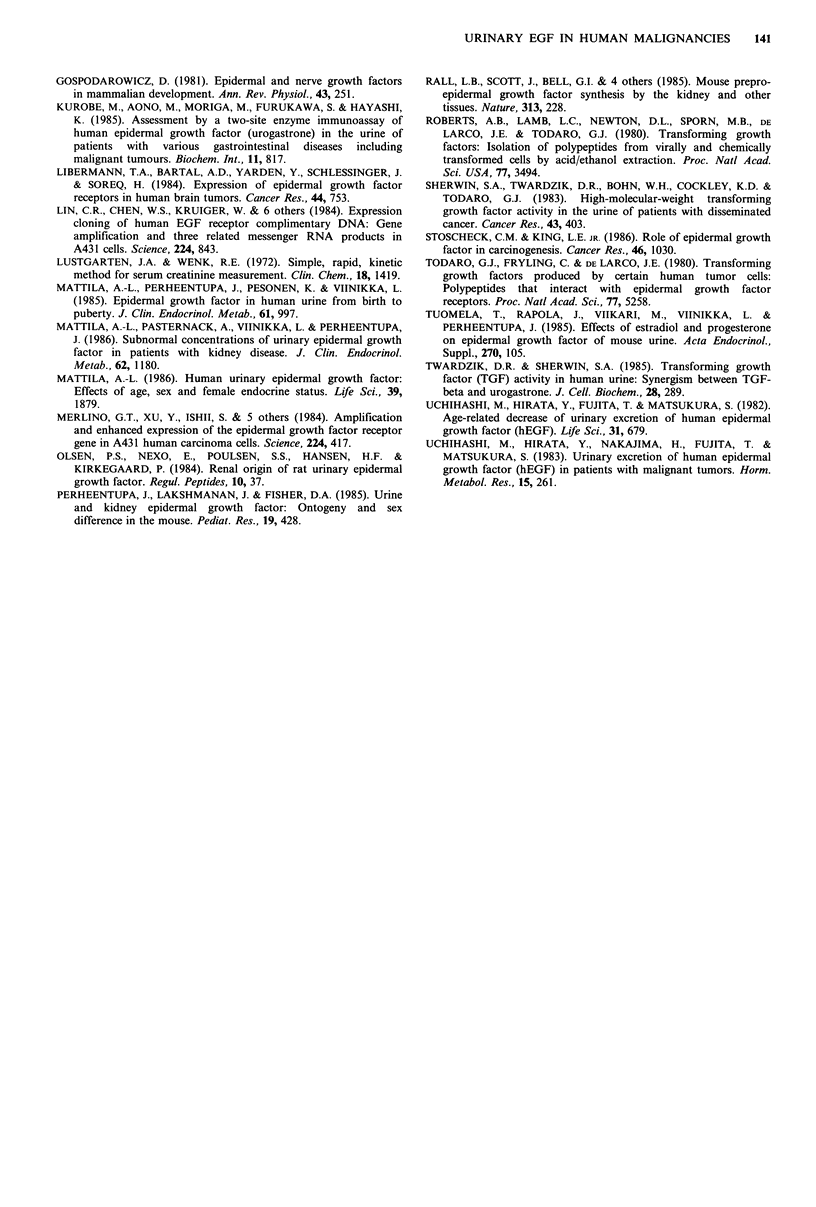# Urinary epidermal growth factor concentrations in various human malignancies.

**DOI:** 10.1038/bjc.1988.29

**Published:** 1988-02

**Authors:** A. L. Mattila, I. Saario, L. Viinikka, O. Ylikorkala, J. Perheentupa

**Affiliations:** Children's Hospital, Helsinki, Finland.

## Abstract

We determined the concentrations of immunoreactive epidermal growth factor in the urine (U-irEGF) of 97 adult patients with various malignancies, including carcinomas of the urinary bladder, kidney, stomach, colon, rectum, breast, endometrium, uterine cervix, ovary, vagina, prostate, pancreas and thyroid, liposarcoma and skin melanoma. The relative U-irEGF concentrations (ng m-1 creatinine) were higher (P = 0.002) for the whole series of female patients than for healthy controls matched for sex and age. Such difference did not appear for male patients. The only specific group with a statistically supranormal U-irEGF concentration (P = 0.0005) comprised women with endometrial carcinoma of the uterus.


Br. J. Cancer (1988), 57, 139–141 © The Macmillan Press Ltd., 1987

### Urinary epidermal growth factor concentrations in various human malignancies

A.-L. Mattila[1], I. Saario[2], L. Viinikka[1], O. Ylikorkala[3] & J. Perheentupa[1]

[1]*Children's Hospital;* [2]*Second Department of Surgery; and* [3]*Department of Obstetrics and Gynaecology, University of Helsinki, 00290 Helsinki, Finland.*

**Summary** We determined the concentrations of immunoreactive epidermal growth factor in the urine (U-irEGF) of 97 adult patients with various malignancies, including carcinomas of the urinary bladder, kidney, stomach, colon, rectum, breast, endometrium, uterine cervix, ovary, vagina, prostate, pancreas and thyroid, liposarcoma and skin melanoma. The relative U-irEGF concentrations (ng m$^{-1}$ creatinine) were higher ($P=0.002$) for the whole series of female patients than for healthy controls matched for sex and age. Such difference did not appear for male patients. The only specific group with a statistically supranormal U-irEGF concentration ($P=0.0005$) comprised women with endometrial carcinoma of the uterus.

Epidermal growth factor (EGF) appears to be involved in the regulation of proliferation, differentiation and differentiated functions of a multitude of cell types (Gospodarowicz, 1981; Carpenter & Zendegui, 1986). In certain normal cells *in vitro* EGF elicits transformation-associated responses (Stoscheck *et al.*, 1986). There is also a considerable body of evidence associating EGF receptor with oncogenesis. The EGF receptor is the product of the c-*erb B* proto-oncogene, which is closely related to the v-*erb B* (Downward *et al.*, 1984). The receptor is present in enormous numbers on the cells of many human epidermoid and glial malignancies (Lin *et al.*, 1984; Merlino *et al.*, 1984; Libermann *et al.*, 1984). Many malignant cells produce another EGF receptor agonist, transforming growth factor alpha (TGF-α) (Todaro *et al.*, 1980; Roberts *et al.*, 1980). TGF-α is excreted in urine by patients with various malignancies (Sherwin *et al.*, 1983), but not by healthy subjects (Twardzik *et al.*, 1985).

Uchihashi *et al.* (1983) and Kurobe *et al.* (1985) found urinary excretion of EGF to be higher in patients with cancer than in healthy persons. This has not been generally accepted. We have now measured urinary immunoreactive EGF (U-irEGF) levels in patients with a variety of malignancies before and after tumour removal.

### Materials and methods

### *Subjects and samples*

Spot urine samples were collected from 97 patients (49 men and 48 women, aged 28–88 years) with various malignancies before surgical removal (or any other treatment) of the tumour. In addition to those shown in Figure 1, six patients had carcinomas, 1 of the vagina, 2 of the pancreas, and 3 of the thyroid, and 1 patient had liposarcoma and 1 skin melanoma. The malignancies included both metastasized and *in situ* tumours. We also measured U-irEGF concentration in 24 patients daily during the first week after removal of the tumour, and in a further 7 patients on the sixth post-operative day. For comparison, urine samples were collected from age- and sex-matched healthy subjects (56 men and 62 women, aged 28–86 years).

### *Assay procedures*

Human EGF, used both as standard and labelled tracer, and a rabbit antiserum against it (8C-217, 3129) were donated by AMGen (Thousand Oaks, California). U-irEGF was measured by a specific homologous radioimmunoassay (Mattila *et al.*, 1985) and creatinine by the kinetic method of Jaffe (Lustgarten & Wenk, 1972).

Correspondence: A.-L. Mattila.
Received 19 March 1987; and in revised form, 1 August 1987.

### *Gel exclusion chromatography*

To determine the molecular size of the U-irEGF we used high-performance gel exclusion chromatography (HPLC) on a prepacked 7.5×300 mm blue column TSK G 2000 SW (LKB, Bromma, Sweden) and an LKB 2150 HPLC pump with a Rheodyne injector, model 7125 (Rheodyne, CA), equipped with a 100-μl sample loop. The column was equilibrated and eluted at room temperature with 0.01 M sodium phosphate buffer (pH 6.8) containing 0.1 M NaCl and 20% acetonitrile (Rathburn HPLC grade S, Walkerburn, Scotland).

### *Statistical analysis*

Since U-irEGF concentrations in humans are age- and sex-dependent (Uchihashi *et al.*, 1982; Mattila, 1986), they were expressed as standard deviation score (SDS), i.e. deviation in SD units from the mean value of age- and sex-matched controls. Because of positive skewness of the distributions of U-irEGF values, all calculations were made after logarithmic transformation. Tests used were Kruskall–Wallis *H*-test, and simple linear correlation and regression analysis (Dixon, 1981).

### Results

To eliminate the effect of variability in the rate of water excretion, U-irEGF concentrations were expressed in ng per mg creatinine, as previously established (Dailey *et al.*, 1978; Mattila *et al.*, 1985). These relative U-irEGF concentrations of the whole series of female cancer patients were higher ($P=0.002$) than those of healthy female controls No such difference appeared for the male cancer patients (Figure 2). The only specific group with a mean U-irEGF concentration higher than in controls, SDS being $+1.3\pm0.3$ (mean ± s.e.m.; $P=0.0005$, Figure 1), comprised women with endometrial carcinoma. In no other group did the size and spread of the tumour seem to influence U-irEGF concentration.

In the women with endometrial carcinoma the relative U-irEGF concentration did not change significantly after tumour removal ($29.3\pm4.2$ *vs.* $25.7\pm3.5$ ng mg$^{-1}$ creatinine, mean ± s.e.m.). Of the female patients with conditions other than endometrial carcinoma, we had a postoperative sample only from 6. The concentration did not change significantly in any other specific group. Patients with renal carcinoma were an exception; unilateral nephrectomy caused ~50% decrease in their relative U-irEGF concentrations.

Gel exclusion chromatography revealed no abnormality in the apparent molecular nature of the urinary irEGF in patients with endometrial carcinoma. Determined in 4

**Figure 1** SD scores (deviation in SD units from the mean value for sex and age) of urinary immunoreactive epidermal growth factor concentrations in individual patients (•, females; *, males) with different malignancies. The hatched areas indicate 95% confidence intervals of the mean values for each group. The age range of the patients is given in parentheses. The *P* value for significant difference from age- and sex-matched controls (SD score 0) is given.

**Figure 2** Regression lines of urinary concentration (ng mg$^{-1}$ creatinine) of immunoreactive epidermal growth factor *vs*. age in female (FP) and male cancer patients (MP), and in female (FC) and male controls (MC). The difference between FP and FC is significant ($P=0.002$).

patients a mean of 95% of the immunoreactivity coeluted with the EGF standard. The rest consisted of high-molecular weight forms of ~20 and 70 kilodaltons.

### Discussion

Urinary irEGF concentrations were statistically supranormal in female cancer patients in general, but as far as specific patient groups were concerned, only in women with endometrial carcinoma. In contrast, Uchihashi *et al.* (1983) found statistically supranormal urinary immunoreactive EGF excretion in patients with carcinomas of the lung, maxilla, oesophagus, stomach, thyroid, breast, and cervix, and in patients with leukaemia, malignant lymphoma and multiple myeloma. Likewise, Kurobe *et al.* (1985) found supranormal urinary EGF levels in patients with gastric carcinoma. Neither series included patients with endometrial carcinoma.

Urinary EGF originates mostly from the kidneys (Rall *et al.*, 1985; Olsen *et al.*, 1984; Mattila *et al.*, 1986). Thus an increase in urinary EGF concentration could be due to stimulation of renal EGF production by some tumour-associated factor. Alternatively, the malignant tumour might produce irEGF, which is excreted via blood to urine. The fact that the U-irEGF decreased by ~50% after unilateral nephrectomy, is an expected consequence of its renal origin.

Since the relative U-irEGF concentrations in the patients with endometrial carcinoma did not decrease after removal of the tumour, it is unlikely that the cancer produced or stimulated the production of the excess of U-irEGF. This excess might rather be another result of some carcinogenic factor(s) affecting these patients. Development of endometrial carcinoma has been suggested to be associated with a preceding supranormal oestrogen production and/or relative progesterone deficiency (Gambrell, 1986). Female sex steroids do appear to regulate urinary irEGF excretion. In both human beings and mice, females excrete more irEGF than males (Uchihashi *et al.*, 1982; Mattila, 1986; Perheentupa *et al.*, 1985). Moreover, in mice oestrogen treatment increased and progesterone treatment decreased U-irEGF concentration (Tuomela *et al.*, 1985).

The statistically supranormal U-irEGF in the whole series of female patients is most interesting, but we have no explanation for this. Evidently it is associated with the fact that 22 of the 27 patients with female-specific carcinoma had values above age- and sex-specific mean values of controls. Against this background the case of endometrial carcinoma is not unique. Perhaps it was by chance that, of the groups with female specific tumours, only this category reached statistical significance.

In conclusion, U-irEGF concentration was statistically supranormal in the whole series of female cancer patients, but in groups with specific malignancies only in patients with endometrial carcinoma. The mechanism of this supranormal excretion is unknown, but it may be hormonally mediated.

We thank Anne Vikman and Hilkka Salmi for skilful technical assistance and Jean Margaret Perttunen for revision of the language. Human EGF and its antiserum were gifts from AMGen (Thousand Oaks, CA).

### References

CARPENTER, G. & ZENDEGUI, J.G. (1986). Epidermal growth factor, its receptor, and related proteins. *Exp. Cell. Res.*, **164**, 1.

DAILEY, G.E., KRAUS, J.W. & ORTH, D.N. (1978). Homologous radioimmunoassay for human epidermal growth factor (urogastrone). *J. Clin. Endocrinol. Metab.*, **46**, 929.

DIXON, W.J. (1981). *BMDP Statistical Software*, University of California Press: Berkeley.

DOWNWARD, J., YARDEN, Y., MAYES, E. & 6 others (1984). Close similarity of epidermal growth factor receptor and v-erb-B oncogene protein sequences. *Nature* (*Lond.*), **307**, 521.

GAMBRELL, R.D. JR.(1986). The role of hormones in the etiology and prevention of endometrial cancer. In *Clinics in Obstetrics and Gynaecology. Endometrial Cancer,* Creasman, W.T. (ed) vol. **13**, p. 695. W.B. Saunder: Philadelphia.

GOSPODAROWICZ, D. (1981). Epidermal and nerve growth factors in mammalian development. *Ann. Rev. Physiol.*, **43**, 251.

KUROBE, M., AONO, M., MORIGA, M., FURUKAWA, S. & HAYASHI, K. (1985). Assessment by a two-site enzyme immunoassay of human epidermal growth factor (urogastrone) in the urine of patients with various gastrointestinal diseases including malignant tumours. *Biochem. Int.*, **11**, 817.

LIBERMANN, T.A., BARTAL, A.D., YARDEN, Y., SCHLESSINGER, J. & SOREQ, H. (1984). Expression of epidermal growth factor receptors in human brain tumors. *Cancer Res.*, **44**, 753.

LIN, C.R., CHEN, W.S., KRUIGER, W. & 6 others (1984). Expression cloning of human EGF receptor complimentary DNA: Gene amplification and three related messenger RNA products in A431 cells. *Science*, **224**, 843.

LUSTGARTEN, J.A. & WENK, R.E. (1972). Simple, rapid, kinetic method for serum creatinine measurement. *Clin. Chem.*, **18**, 1419.

MATTILA, A.-L., PERHEENTUPA, J., PESONEN, K. & VIINIKKA, L. (1985). Epidermal growth factor in human urine from birth to puberty. *J. Clin. Endocrinol. Metab.*, **61**, 997.

MATTILA, A.-L., PASTERNACK, A., VIINIKKA, L. & PERHEENTUPA, J. (1986). Subnormal concentrations of urinary epidermal growth factor in patients with kidney disease. *J. Clin. Endocrinol. Metab.*, **62**, 1180.

MATTILA, A.-L. (1986). Human urinary epidermal growth factor: Effects of age, sex and female endocrine status. *Life Sci.*, **39**, 1879.

MERLINO, G.T., XU, Y., ISHII, S. & 5 others (1984). Amplification and enhanced expression of the epidermal growth factor receptor gene in A431 human carcinoma cells. *Science*, **224**, 417.

OLSEN, P.S., NEXO, E., POULSEN, S.S., HANSEN, H.F. & KIRKEGAARD, P. (1984). Renal origin of rat urinary epidermal growth factor. *Regul. Peptides*, **10**, 37.

PERHEENTUPA, J., LAKSHMANAN, J. & FISHER, D.A. (1985). Urine and kidney epidermal growth factor: Ontogeny and sex difference in the mouse. *Pediat. Res.*, **19**, 428.

RALL, L.B., SCOTT, J., BELL, G.I. & 4 others (1985). Mouse prepro-epidermal growth factor synthesis by the kidney and other tissues. *Nature*, **313**, 228.

ROBERTS, A.B., LAMB, L.C., NEWTON, D.L., SPORN, M.B., DE LARCO, J.E. & TODARO, G.J. (1980). Transforming growth factors: Isolation of polypeptides from virally and chemically transformed cells by acid/ethanol extraction. *Proc. Natl Acad. Sci. USA*, **77**, 3494.

SHERWIN, S.A., TWARDZIK, D.R., BOHN, W.H., COCKLEY, K.D. & TODARO, G.J. (1983). High-molecular-weight transforming growth factor activity in the urine of patients with disseminated cancer. *Cancer Res.*, **43**, 403.

STOSCHECK, C.M. & KING, L.E. JR. (1986). Role of epidermal growth factor in carcinogenesis. *Cancer Res.*, **46**, 1030.

TODARO, G.J., FRYLING, C. & DE LARCO, J.E. (1980). Transforming growth factors produced by certain human tumor cells: Polypeptides that interact with epidermal growth factor receptors. *Proc. Natl Acad. Sci.*, **77**, 5258.

TUOMELA, T., RAPOLA, J., VIIKARI, M., VIINIKKA, L. & PERHEENTUPA, J. (1985). Effects of estradiol and progesterone on epidermal growth factor of mouse urine. *Acta Endocrinol.*, Suppl., **270**, 105.

TWARDZIK, D.R. & SHERWIN, S.A. (1985). Transforming growth factor (TGF) activity in human urine: Synergism between TGF-beta and urogastrone. *J. Cell. Biochem.*, **28**, 289.

UCHIHASHI, M., HIRATA, Y., FUJITA, T. & MATSUKURA, S. (1982). Age-related decrease of urinary excretion of human epidermal growth factor (hEGF). *Life Sci.*, **31**, 679.

UCHIHASHI, M., HIRATA, Y., NAKAJIMA, H., FUJITA, T. & MATSUKURA, S. (1983). Urinary excretion of human epidermal growth factor (hEGF) in patients with malignant tumors. *Horm. Metabol. Res.*, **15**, 261.